# A Novel Rat Model of Orthodontic Tooth Movement Using Temporary Skeletal Anchorage Devices: 3D Finite Element Analysis and *In Vivo* Validation

**DOI:** 10.1155/2014/917535

**Published:** 2014-09-10

**Authors:** Neelambar Kaipatur, Yuchin Wu, Samer Adeeb, Thomas Stevenson, Paul Major, Michael Doschak

**Affiliations:** ^1^Division of Orthodontics, University of Alberta, Edmonton, AB, Canada T6G 2E1; ^2^Biomedical Engineering, University of Alberta, Edmonton, AB, Canada T6G 2E1; ^3^Civil Engineering, University of Alberta, Edmonton, AB, Canada T6G 2E1; ^4^Department of Dentistry, University of Alberta, Edmonton, AB, Canada T6G 2E1; ^5^Faculty of Pharmacy & Pharmaceutical Sciences, University of Alberta, Edmonton, AB, Canada T6G 2E1; ^6^Pharmaceutical Orthopaedic Research Lab, 2-020J Katz Group Centre for Pharmacy & Health Research, University of Alberta, 11361-87 Avenue, Edmonton, AB, Canada T6G 2E1

## Abstract

The aim of this animal study was to develop a model of orthodontic tooth movement using a microimplant as a TSAD in rodents. A finite element model of the TSAD in alveolar bone was built using *μ*CT images of rat maxilla to determine the von Mises stresses and displacement in the alveolar bone surrounding the TSAD. For* in vivo* validation of the FE model, Sprague-Dawley rats (*n* = 25) were used and a Stryker 1.2 × 3 mm microimplant was inserted in the right maxilla and used to protract the right first permanent molar using a NiTi closed coil spring. Tooth movement measurements were taken at baseline, 4 and 8 weeks. At 8 weeks, animals were euthanized and tissues were analyzed by histology and EPMA. FE modeling showed maximum von Mises stress of 45 Mpa near the apex of TSAD but the average von Mises stress was under 25 Mpa. Appreciable tooth movement of 0.62 ± 0.04 mm at 4 weeks and 1.99 ± 0.14 mm at 8 weeks was obtained. Histological and EPMA results demonstrated no active bone remodeling around the TSAD at 8 weeks depicting good secondary stability. This study provided evidence that protracted tooth movement is achieved in small animals using TSADs.

## 1. Introduction

Orthodontic tooth movement (OTM) occurs through controlled application of mechanical forces on teeth and surrounding biological tissues [1]. Current rat models of OTM (for molar mesialization) utilize the maxillary incisors for anchorage and employ a NiTi closed coil spring attached to the molar tooth to deliver a specific magnitude of force [2–4]. This model is currently used in rodent research due to easy accessibility to secure the appliance but has several disadvantages including retardation in normal eruption process of incisor [5], loss of pulp vitality of the incisor [3, 5], and change in force vector due to continuous incisor eruption that occurs in rats, with subsequent loss of anchorage [6]. Hence, it would be advantageous to develop an anchorage device that is easily inserted, provides stable anchorage, and maintains a constant force delivery without the undesirable side effects mentioned above.

Mini-implants have been used extensively as stable anchorage devices to achieve predictable tooth movement [7–9]. Although there are numerous commercial mini-implant systems available for use in humans, they remain too large for application to the rat. An alternative approach is to use microimplants, as their miniature size allows them to be placed in practically any location and are similar to those used for osteotomy fixation during orthognathic surgery and facial reconstructions [10–12].

In the current study, we used a Stryker titanium microimplant (1.2 × 3 mm in diameter) (Stryker-Leibinger Inc., Hamilton, ON, Canada) as a temporary skeletal anchorage device (TSAD) because of its smaller size and availability. In order for the TSAD to withstand forces of magnitude large enough to cause tooth movement, it would likely result in loading of surrounding cortical and cancellous bone in which it is inserted. The effects upon surrounding alveolar bone during TSAD placement and subsequent anchorage for tooth movement have not been studied extensively and the effect of those stresses on bone remodeling remains unanswered. Thus, we chose to employ the finite element method (FEM) as a tool to define stress concentrations on the surrounding bone during post insertion anchorage for tooth movement.

FEM is a numerical method of analyzing stresses and deformations in any structure of a given geometry. The structure geometry (precise or imprecise) is discretized into so called “finite elements” connected to each other by nodes. The type, arrangement, and total number of elements affect the accuracy of the results. FEM has become the most used computational and analysis tool since the 1960's and was first used in implant dentistry in 1976 [13]. It is postulated that a TSAD inserted into the alveolar bone changes the local stress state of the bone and induces an adaptive phenomena. Stress distribution depends on many assumptions including geometry of the model studied, material properties of the bone and the TSAD, boundary conditions, and load applied, along with contact status between the TSAD and surrounding cortical bone [14]. Currently, with the advent of advanced imaging techniques and improvement in mathematical computation methods, a precise geometric representation of the actual model can be considered for near accurate results [15].

An ideal animal model for OTM should include a force system with constant magnitude of force in the desired direction and provide sufficient anchorage to trigger tooth movement without any undesirable side-effects. Therefore, the objective of the present study was to develop an Finite Element (FE) model of a TSAD in the rat maxilla to estimate the stress distribution in the surrounding cortical bone and the TSAD stability at different force levels followed by* in vivo* validation using a rodent model of OTM.

## 2. Materials and Methods

### 2.1. Building an FE Model

The FEM was built as follows. Microcomputed tomography (*μ*CT) images of the rat maxilla were obtained from scanned data using Skyscan 1076 imager for small animals. (SkyScan 1076, Kontich, Belgium) The images were imported into Mimics (Mimics 13.1, Leuven, Belgium) to segment the maxilla by Hounsfield values and manual mask segmentation. Three-dimensional geometry files were created for each mask and saved as stereolithography (STL) files. Computer Assisted Design (CAD) software (Geomagic 12.0, Research Triangle Park, NC, USA) was used for extracting surfaces and solids from STL files. Triangle and intersection fixing techniques were performed and then Standard for the Exchange of Product model data (STEP) files were created and exported for the maxilla and molar teeth separately into ABAQUS. ABAQUS, FE modeling software (ABAQUS 6.9.1, Providence, RI, USA) with CAE and Solver modules, was used for pre- /postprocessing and analysis calculation. The geometry of the TSAD was created in ProE (Pro/Engineer Needham, MA, USA) according to exact dimensions of the actual Stryker 1.2 × 3 mm microimplant and imported into ABAQUS. The TSAD was registered at the desired location on the rat maxilla based on the amount of bone present and insertion depth required. The inserted depth of the TSAD was approximately 1.5 mm from the cortical surface of the bone to the bottom tip of the TSAD. This depth of 1.5 mm was based on the thickness of the maxillary bone in the proposed location of TSAD placement as measured by *μ*CT.

**Figure 1:**
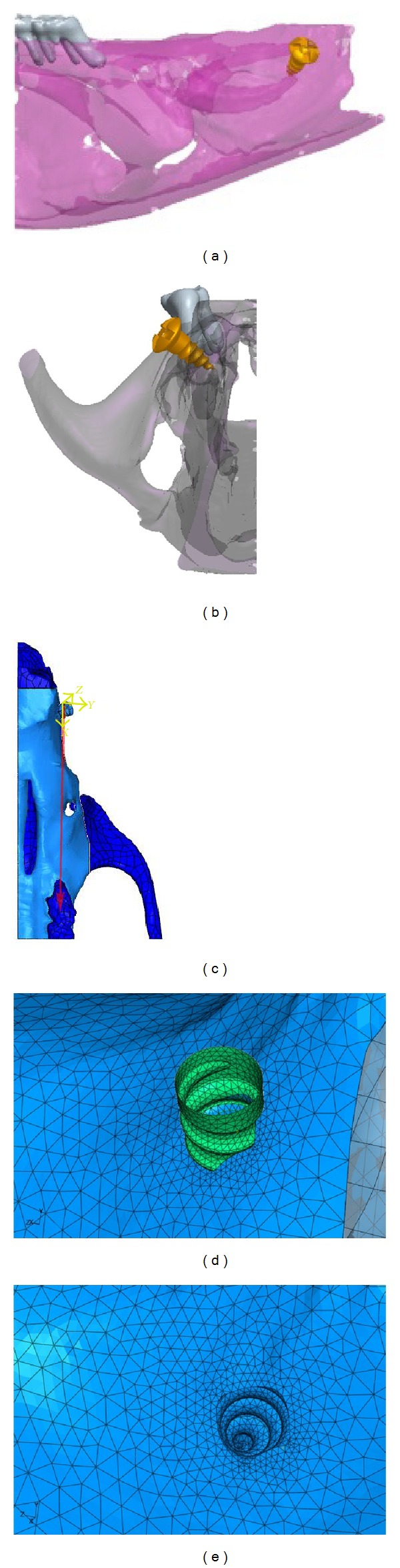
Computer modeling of TSAD placement into the rat maxilla 3D model of hemimaxillae in (a) sagittal view and (b). Transverse view (c). 3D model of the hemimaxillae showing the direction of force applied during FE analysis (d), (e). Fine mesh of the TSAD and the surrounding maxillary bone.

Material properties of the rat maxillary bone (Young's modulus (*ε*) −20.0 Gpa; Poisson's ratio (*ν*) −0.3) were obtained from literature [16]. The TSAD was modeled as a rigid body as it was assumed that the titanium implant with a high Young's modulus would not undergo any measurable deformation at the force level applied in this study. A reference point (RP) was defined just anterior to the TSAD to represent the motion of the rigid body. The maxilla was meshed as 10-node tetrahedron elements, C3D10M; for proper contact performance and to model the threads of the TSAD appropriately, a smaller element size (0.1 mm) was used to model them locally. Contact was set between the TSAD and the maxilla, so that small sliding was allowed between the contact surfaces. Contact between the TSAD and alveolar bone was considered friction affected and friction coefficient was set at 0.2 [16]. Constraints (boundary conditions) were applied to the maxilla on the mesial end to allow for bone bending and displacement in the direction of the load. Once the material properties and boundary conditions were assigned, the force was applied on RP in the direction of the first permanent molar to mimic the direction of force applied during actual tooth movement. The model was analyzed by ABAQUS processor and postprocessing results were displayed in the form of color-coded maps of von Mises stresses and displacements of the alveolar bone around the TSAD (Figure 1).

### 2.2. Animal Model of OTM

**Figure 2:**
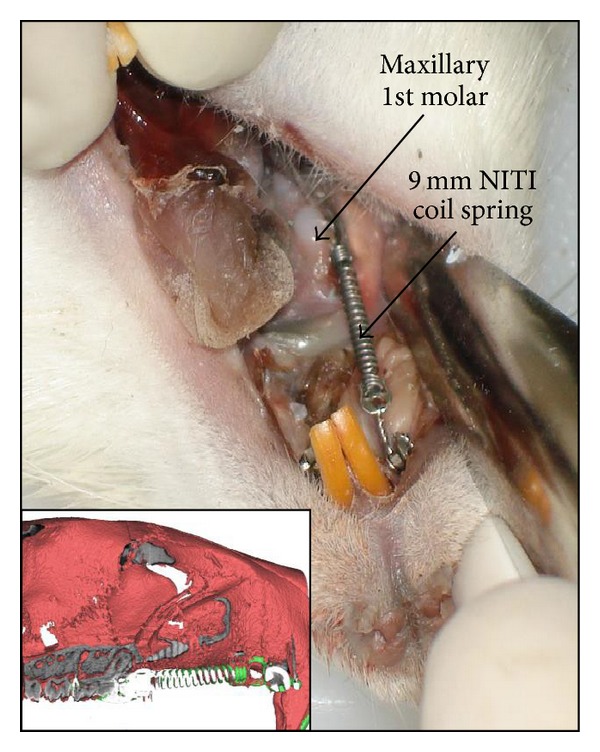
Orthodontic appliance in the right maxilla for tooth movement using TSAD and NiTi closed coil spring. Inset: *μ*CT 3D rendered cross-section model of rat maxilla with appliance.

Ethics approval was obtained from the animal care and use committee of the University of Alberta. Three-month-old female Sprague-Dawley rats (*n* = 25) were obtained from Biosciences, University of Alberta and caged in animal housing with 12 hours dark and light cycles and fed a soft diet* ad libitum*. Rats were sedated using general anesthesia 2% Isoflurane/L oxygen (Forane, Baxter, Deerfield, IL, USA) and placed supine in a custom designed surgical jig (i.e., respiratory plenum). To insert the temporary skeletal anchorage device, a 4 mm semilunar incision was made from the distopalatal gingival margin of the maxillary right incisor posteriorly with a number 15 surgical blade. After achieving adequate hemostasis, a pilot hole was drilled in the maxillary bone at a 45° angle using a 0.5 mm round bur attached to a NSK slow speed electric dental handpiece. (NSK; Brassler, Savannah, GA, USA) A self-threading Stryker 1.2 × 3 mm titanium TSAD (Stryker-Leibinger, Hamilton, ON, Canada) was inserted to a depth of 1.5 mm into the alveolar bone about 12 to 14 mm distance from the mesial aspect of the right first permanent molar. The fit and primary stability of the TSAD into the alveolar bone was verified by finger pressure, with a side-to-side and in-and-out motion. A stainless steel ligature wire was placed around the neck of the right first permanent molar and secured in position by tightening. A 9 mm closed coil NiTi spring (GAC International, Bohemia, NY, USA), was secured to the posterior molar and the TSAD neck anteriorly with 0.010 inches stainless steel ligature. The appliance was left in place for 8 weeks to achieve appreciable tooth movement, as measured by *μ*CT imaging (described below). The left side acted as an intra-animal control with no appliance (Figure 2).

### 2.3. *μ*CT Analysis of OTM

All rats underwent baseline* in vivo* *μ*CT scan (Skyscan 1076 “*in*-*vivo*” *μ*CT, Skyscan NV, Kontich, Belgium) of the alveolar bone surrounding the first molar and extending anteriorly to the maxillary incisors. The scans were repeated* in vivo* after 4 weeks and 8 weeks of appliance placement and tooth movement was accurately measured from *μ*CT projections using bundled vendor analysis software (DataViewer, Skyscan, Kontich BE). For all *μ*CT imaging, scans were conducted at 100 kV and 100 mA current through 180° with a rotation step of 0.5° to produce serial projectional images of isotropic 18 *μ*m^3^ voxels. All image data was processed using commercial software bundled with the *μ*CT system in our laboratory. The acquired data was Gaussian filtered and underwent global thresholding to extract the mineralized phase representing the 3D tooth movement and bone architecture. Measurements were made between the first and second right maxillary molars at 0, 4, and 8 weeks.

### 2.4. Measurement of Tooth Movement and TSAD Displacement

The measurement of amount of tooth movement and TSAD displacement was accomplished as follows. Briefly, microcomputed tomography-rendered 2-dimensional scans were reconstructed as  .bmp files and viewed using Data Viewer software (DataViewer, Skyscan, Kontich BE). The 2-dimensional slices displayed as 3 orthogonal sections in the *x*, *y*, and *z* planes of space were centered at the desired point inside the reconstructed space. Once the image was centered in all 3 planes, the linear distance from the most convex contact area between the maxillary right first and second molars was measured and recorded for the amount of tooth movement. For the TSAD displacement, the linear distance from the TSAD head to centre of the right third permanent molar was measured and recorded. Measurements were recorded at 0, 4, and 8 weeks, and the amount of tooth movement and TSAD displacement was obtained by subtracting the distances at 4 and 8 weeks from the baseline. Measurements were obtained by the primary author (Neelambar Kaipatur) in a blinded fashion and repeated for reliability one week apart (*r* = 0.96).

### 2.5. Electron Probe Microanalysis (EPMA)

All animals were given a brief 10-day pulse of elemental strontium (Strontium ranelate, PROTOS; Servier Laboratories, Hawthorn, Victoria, Australia; 308 mg/kg/day body weight—subtherapeutic dosage at lower limit of therapeutic index) by gavage; 10 days prior to euthanization. Strontium has been shown as an excellent dynamic label for bone turnover and can readily be detected by EPMA (Electron-Probe Microanalysis) at high spatial resolution [17]. EPMA was performed on the palatal half of the right first permanent molar and around the TSAD for spatially mapping the location and distribution of elemental, Strontium (Sr), Calcium (Ca), and Phosphate (P). Briefly, the sagittal sections of right first molar and the alveolar bone surrounding the TSAD were defatted in acetone, embedded in epoxy resin, progressively polished (~0.5 *μ*m), and scanned at 2 and 5 *μ*m resolutions to qualitatively analyze the Ca, Sr, and P content in the alveolar bone surrounding the TSAD, and the molars.

### 2.6. Histological Assessment

At the experiment end point (8 weeks), all animals were euthanized using isoflurane followed by CO_2_ inhalation to effect, and the right and left maxilla were immediately dissected, stored in 4% paraformaldehyde (Sigma-Aldrich Canada, Oakville, ON, Canada) and fixed for 1 week with frequent changes. Following fixation, each hemimaxilla was cut sagittally at the level of first permanent molar. The palatal half was processed for spatial mapping of bone turnover using EPMA and the buccal half was processed for routine histology. The alveolar bone surrounding the TSAD was also processed. All samples processed for histology were rinsed with PBS (phosphate buffered saline) wash buffer (pH 7.3) and immersed in 4.13% EDTA (disodium ethylene diamine tetra acetic acid; Sigma-Aldrich Canada, Oakville, ON, Canada) decalcifying solution for 3 weeks. The tissue was checked and further decalcified if inadequate decalcification was observed. Following decalcification, samples were processed for routine histology by paraffin embedding. Sagittal sections (6 *μ*m) were cut and stained with hematoxylin and eosin for routine histology.

### 2.7. Statistical Analysis

SPSS statistical software (version 16.0; SPSS, Chicago, IL, USA) was used to analyze the data. OTM measurements and TSAD displacement obtained from age-matched cohorts were used for statistical analyses. All quantitative data were expressed as mean ± standard error (SE). To compare the mean amount of tooth movement and mean TSAD displacement at 4 and 8 weeks, repeated measures ANOVA were performed with significance level set at 95% (*α* = 0.05). A bonferroni post hoc comparison was performed within groups to see individual variation. Since the results of FE analysis were individual results without a statistical spread, we reported outcome of von Mises stress and displacement at different force levels without performing any statistical comparison to test level of significance.

## 3. Results

### 3.1. FE Analysis

**Figure 3:**
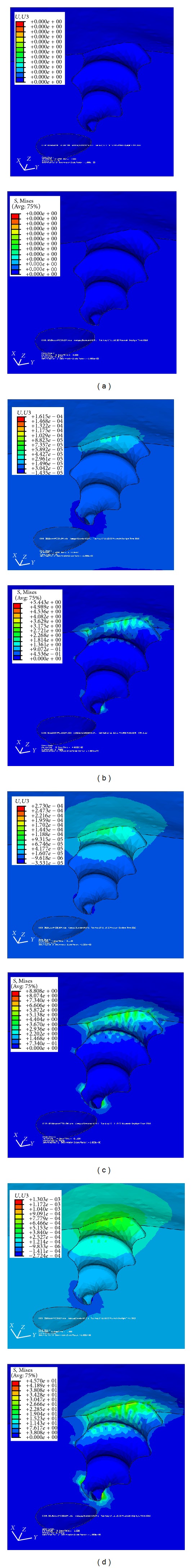
Displacement (panel above) and von Mises stresses (panel below) at (a). 0 gms, (b). 30 gms (c). 60 gms and (d). 140 gms of force. The warmer colors depict increase in the amount and distribution of the stress and displacement and shows stresses concentrated at the apex of the TSAD and at the coronal contact area between the TSAD and the bone.

**Figure 4:**
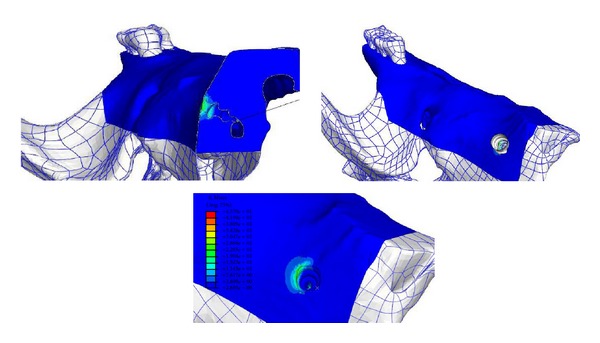
Von Mises stress localization in the cortical bone surrounding the TSAD. Dark blue color represents minimal von Mises stress and red color represents maximum von Mises stress localization around the TSAD.

The FE method was used to predict the von Mises stresses in the cortical bone surrounding the TSADs. The von Mises stress distribution and the resultant displacement of the bone surrounding the TSAD at different force levels are presented in Figure 3. Dark blue color represents areas with minimal von Mises stress and minimal displacement and red color represents area with maximum von Mises stress and maximum displacement with gradient of colors in between. Understanding the limitation of this FE model, with the assigned material properties, friction coefficient, contact, and boundary conditions, the maximum von Mises stresses on the contact surface of bone was 45.7 MPa, near the apex of the TSAD, but the majority of the stresses throughout were under 25 MPa. Finite element analysis revealed that the rat maxillary bone could withstand stress of up to 140 gms force traction on the TSAD toward the molar, with possibly higher stress at the thread edge due to local stress concentration. The micromotion of the TSAD under 140 gms of force was only −0.89 to 0.136 *μ*m along the TSAD axis and 2.29 *μ*m along the force direction. The maximum displacement of the bone was 0.64 *μ*m (Figure 4).

### 3.2. OTM and TSAD Stability

**Figure 5:**
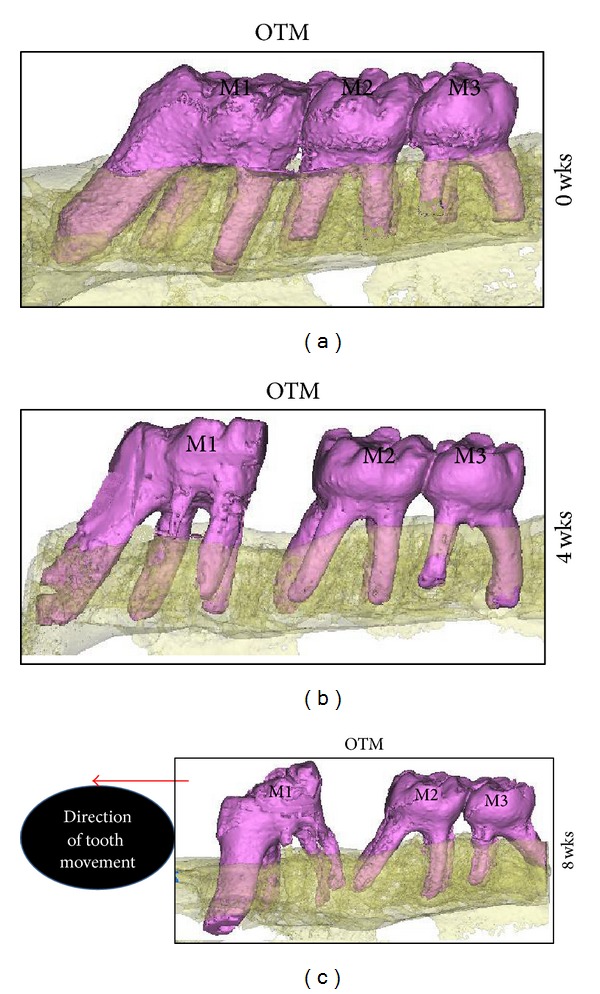
Three-dimensional microcomputed tomography—rendered images showing the amount of tooth movement of the right first permanent molar at 4 and 8 weeks. (M1, right permanent first molar; M2, right permanent second molar; M3, right permanent third molar).

**Figure 6:**
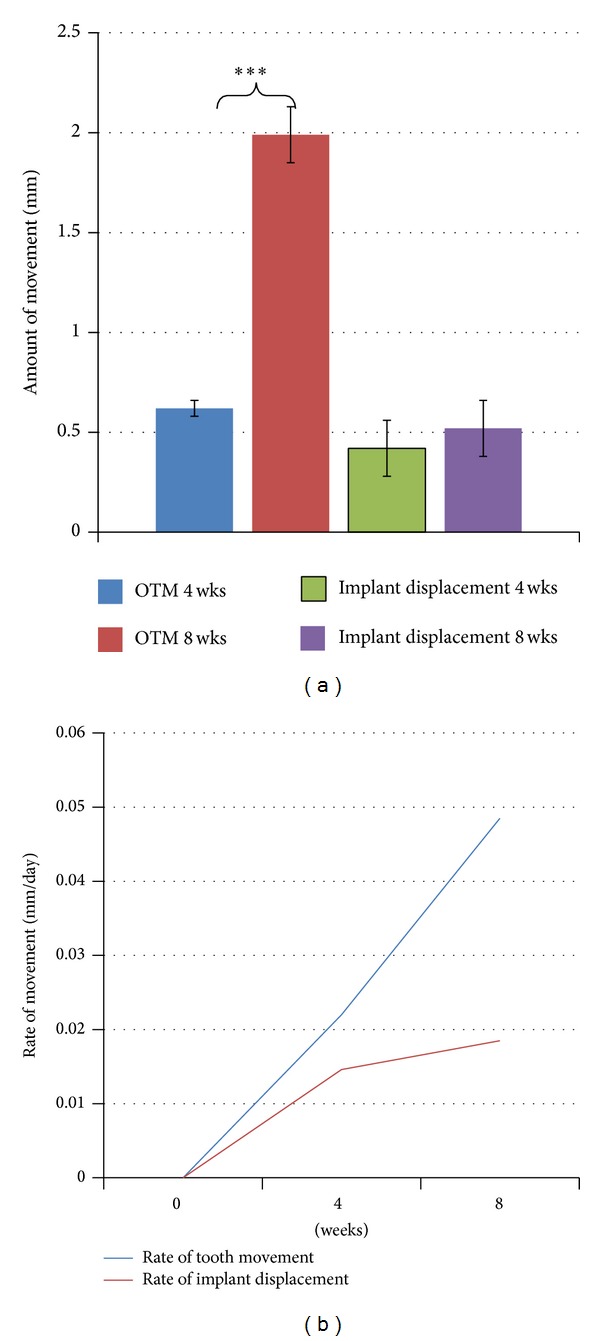
(a) Mean (±S.E) amount of orthodontic tooth movement and implant displacement measured at 4 and 8 weeks. (Significance level: *P < 0.05). (b) Mean rate of orthodontic tooth movement and implant displacement measured at 0, 4, and 8 weeks.

All animals were healthy and gained weight steadily during the entire treatment time with no evidence of significant weight loss. The survival rate of TSADs was 92% at four weeks and 80% at 8 weeks. This was due to loosening of two TSADs between 0 and 4 weeks and three TSADs between 4 and 8 weeks. These animals were removed from the study. Results of tooth movement at 0, 4, and 8 weeks can be seen in the *μ*CT images in Figure 5. Kolgomorov-Smirnnov test for normality and Levene's test for equal variance were satisfied. In terms of measured distance, there was substantial tooth movement, both translation and tipping, at 4 weeks (0.62 ± 0.04) and 8 weeks (1.99 ± 0.14) (Figure 6(a)). Repeated measures ANOVA showed a high statistically significant tooth movement with a *P* value of 0.0003. Pairwise comparisons at both 4 and 8 weeks compared to baseline showed statistically significant tooth movement (*P* ~ 0.0003). The rate of tooth movement was 0.022 mm/day from zero to 4 weeks with a steady increase to 0.048 mm/day from 4 to 8 weeks with statistically significance (*P* ~ 0.0001) TSAD displacement was measured to be 0.42 mm ± 0.14 mm at 4 weeks and 0.94 mm ± 0.17 mm at 8 weeks. The rate of TSAD displacement was 0.014 mm/day from zero to 4 weeks and remained constant with very minimal increase in rate from 4 to 8 weeks (0.018 mm/day) (Figure 6(b)). There was no statistically significant difference in TSAD displacement from baseline to 4 weeks (*P* ~ 0.057) but from 4 weeks to 8 weeks, the amount of TSAD displacement was significant (*P* ~ 0.016). Rate of TSAD displacement was not significant at 4 and 8 weeks (Figure 6(a)).

### 3.3. Elemental Mapping of Ca, P, and Sr in the Alveolar Bone

**Figure 7:**
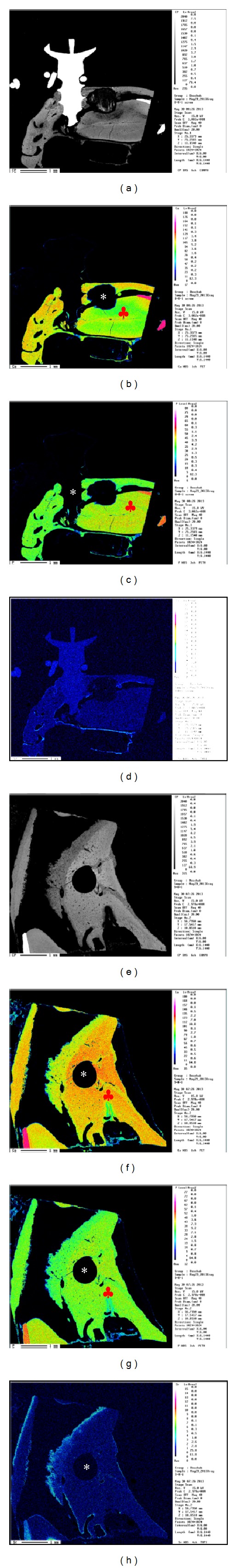
Sagittal (a) and cross-sectional (e) backscattered images and electron microprobe mapping of calcium ((b) and (f)); phosphorus ((c) and (g)); strontium ((d) and (h)) composition of the alveolar bone surrounding the micro-TSAD. No evidence of recognizable difference in the calcium or phosphorus levels of bone immediately around the micro-TSAD and the surrounding preexisting bone (*♣*). ((d) and (h)) show no strontium deposition in the alveolar bone immediately surrounding the micro-TSAD. Lack of strontium deposition confirms no active bone remodeling and excellent micro-TSAD stability. ∗TSAD cavity. Scale bars = 1 mm in ((a)–(h)).

Figure 7 shows EPMA mapped densities of Ca, P, and Sr in the bone around the TSAD. There was no evidence of recognizable difference in the Ca density of bone immediately around the TSAD when compared to that of surrounding distant alveolar bone. (Figures 7(b) and 7(f)) The concentration of P also demonstrated similar findings. (Figures 7(c) and 7(g)). Figures 7(d) and 7(h) show that elemental strontium was not readily detected in the alveolar bone immediately surrounding the TSAD indicating no active bone remodeling around the TSAD.

**Figure 8:**
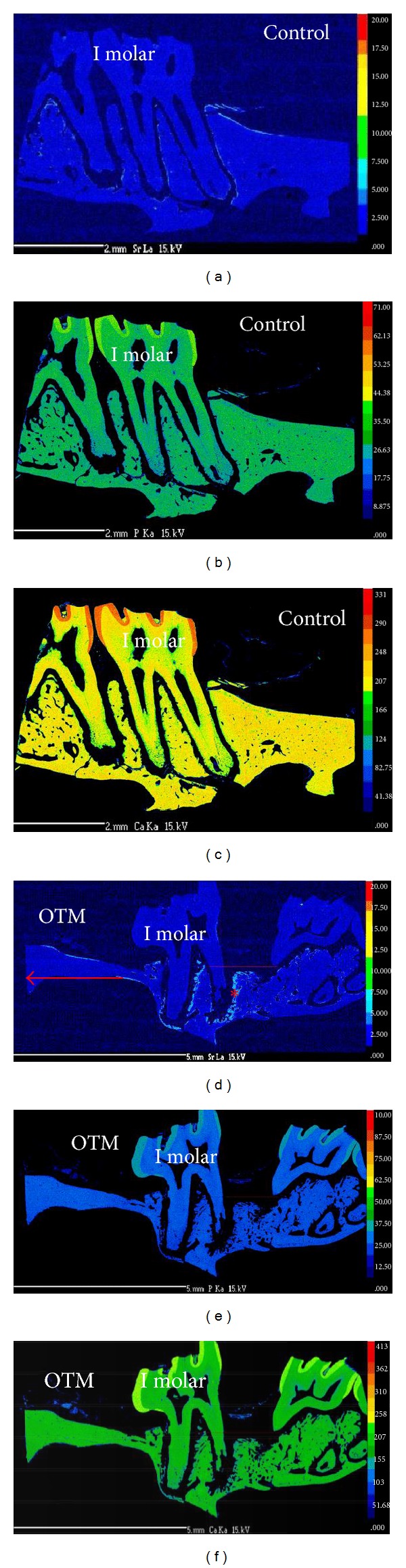
Electron microprobe mapping of strontium ((a) and (d)) calcium ((b) and (e)); phosphorus ((c) and (f)) composition of the alveolar bone surrounding the upper left (control) and right first permanent molar (OTM), respectively. Panel (d) shows increased strontium deposition (∗) surrounding the roots of right first permanent molar where OTM occurred. Evidence of increased strontium deposition indicates increased bone remodeling on the tension side of OTM. No difference in the Ca ((b) and (e)) and P ((c) and (f)) composition could be seen between control and OTM side. OTM-orthodontic tooth movement, ⟵ indicates direction of tooth movement. Scale bars = 2 mm in ((a)–(c)); 5 mm in ((d)–(f)).

We detected increased elemental strontium deposition in newly mineralizing alveolar bone shown as warmer colors on the tension side of tooth movement around the roots of right first permanent molar (Figure 8(d)) indicating robust alveolar bone remodeling associated with orthodontic tooth movement. Minimal or no deposition of Sr was seen on the control side (Figure 8(a)). There was no detectable difference in densities of Ca (Figures 8(b) and 8(e)) and P (Figures 8(c) and 8(f)) found between control and OTM side.

### 3.4. Histological Results

**Figure 9:**
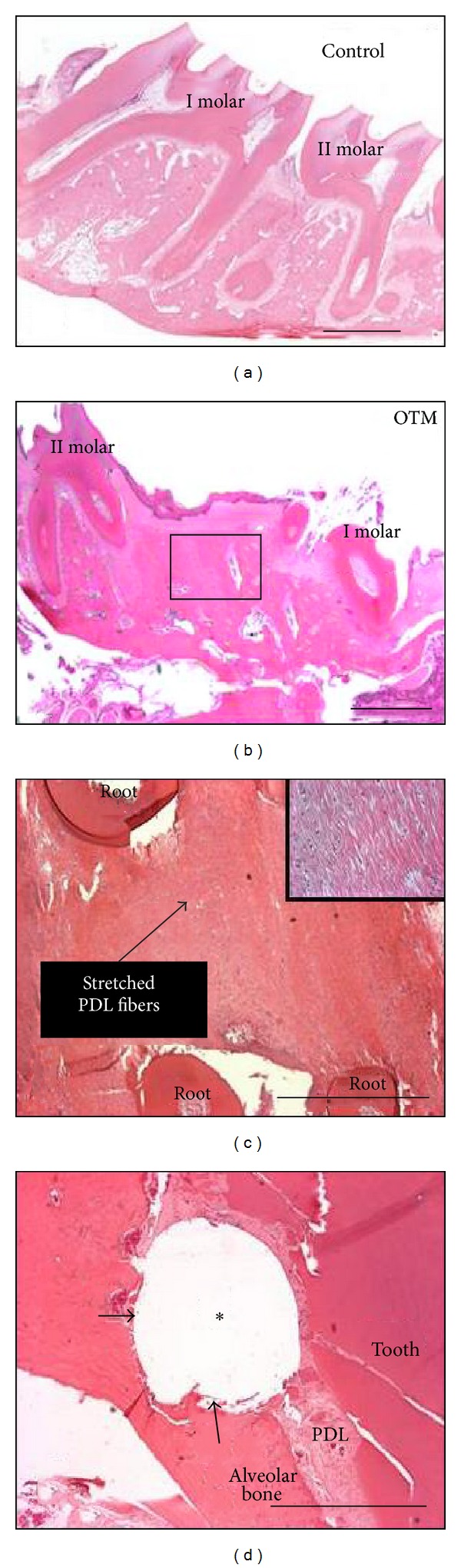
Histologic hematoxylin and eosin stained paraffin sections of the maxillary left (a) and right (b) first permanent molar area. While normal bone remodeling occurs on the control side with no gap between the first and second molar (a), increased separation between the first and second permanent molars (tooth movement) and increased bone remodeling is evident on the tension side of OTM (b). (c) Higher magnification of boxed area in (b) with stretched PDL fibers (Inset). (d) Alveolar bone surrounding the micro-TSAD, (arrows) with part of TSAD inadvertently into PDL space surrounding the tooth root. ∗ denotes TSAD space; scale bars = 1 mm.

Figure 9 shows hematoxylin and eosin stained sections of rat maxilla at level of first molar (Figures 9(a) and 9(b)) and TSAD (Figure 9(d)). While no active remodeling is seen on the control side, robust alveolar bone remodeling with stretching of PDL fibers and enlarged blood vessels is seen on the tension side of right first permanent molar (Figure 9(c)). The alveolar bone surrounding the TSAD had good bone—TSAD contact with no signs of cellular infiltrate (Figure 9(d)). Inadvertent infiltration and damage to the PDL was also seen with this tooth movement model.

## 4. Discussion

Our study effectively used a TSAD as an anchorage device to facilitate OTM in rats. The results showed substantial tooth movement at 4 weeks (0.62 mm) and 8 weeks (1.99 mm) compared to baseline. With the exception of five TSADs (two failed between 0 and 4 weeks and three TSADs became loose between 4 and 8 weeks), the remaining twenty were stable and well integrated with the surrounding alveolar bone during the 8 week experimental period. Our success rate of 92% at 4 weeks and 80% at 8 weeks was comparable to a success rate of 83.6% reported in recent meta-analyses [18, 19]. Various factors affect TSAD stability during insertion and following loading and can be broadly divided into factors affecting primary and secondary stability. Factors affecting primary stability play a role during the first month after insertion, after which factors affecting secondary stability take over. Thickness of overlying gingival tissue, TSAD design, diameter, length, pitch of screw, and distance between threads and micromotion during insertion all affect primary stability [20, 21]. High micromotion coupled with early loading can lead to TSAD loosening and subsequent failure. Literature suggests a critical micromotion level to be between 50 and 150 *μ*m [22]. Although our FE results showed a micromotion of 0.136 *μ*m, the micromotion analyzed in our FE model was during load application and did not take into account micromotion encountered during insertion. Excessive micromotion due to increased insertion torques could lead to microcrack propagation in the alveolar bone resulting in accelerated bone turnover leading to TSAD loosening and failure. We hypothesize that loosening of two TSADs between week 0 and 4 was due to lack of primary stability from excessive micromotion during insertion. The primary author found it very challenging to control insertion torque and minimize micromotion due to the miniature size of the TSAD. Skeggs et al. [23] in their Cochrane review discussed that during TSAD placement, the surgeon should be aware of the depth of the TSAD into the actual bone and not the bone and soft tissue insertion. This is absolutely critical as a thick soft tissue biotype can deceive the clinician from achieving primary stability. In our study 1.5 mm insertion depth of the TSAD into the cortical bone was based on FE analysis wherein minimal displacement of the TSAD was observed (2.24 *μ*m) even at 140 gms of force. The amount and type of force applied is critical for effective tooth movement to avoid critical failures. Bernhart et al. [24] showed that excessive force during orthodontic loading can lead to microfractures and mobility, and light forces [25] can lead to adequate bone remodeling and accelerated stability [26]. Based on the FE results, 140 gms force was the amount that a rat maxillary bone (*σ* = 45.7 MPa) could tolerate without permanent deformation of the bone [27]. Although the maxillary bone could tolerate a force of 140 gms without permanent deformation, the loading force employed in our study using NiTi coil spring was ~30 gms as reported in literature [3] as the amount of force needed to protract a rat molar is very small. Miyawaki et al [8] achieved 85% success rate of microscrews and attributed the 15% failure rate to peri-implant inflammation. Freudenthaler et al. [28] and Roberts et al. [29] supported this view and showed that the most important factors affecting TSAD stability were peri-implant inflammation rather than orthodontic loading. Based on FE results, less than 25 MPa von Mises stress and 0.64 *μ*m displacement was observed, indicating ideal loading force for implant stability. Although we did not measure peri-implant inflammation, we hypothesize that peri-implant inflammation might cause accelerated bone turnover and TSAD loosening. Secondary stability of the TSAD starts about one month after TSAD insertion and depends on bone remodeling around the implant and amount of bone-to-implant contact. For bone remodeling to occur around the TSAD an optimal level of strain should be achieved not exceeding the critical limit of 4000 microstrain [30]. Our FE results showed maximal displacement of the alveolar bone around 0.64 *μ*m. EPMA and histology showed adequate bone-to-implant contact with no signs of active bone turnover as evidenced by lack of strontium deposition in the alveolar bone around the TSAD. Strontium is known to act as a surrogate to calcium during bone remodeling by replacing calcium in the newly forming bone. Lack of strontium deposition around the TSAD suggests no active bone remodeling indicating good secondary stability (Figures 7(d) and 7(h)). There were no changes in the densities of calcium and phosphorus of the bone immediately surrounding the TSAD in comparison to a distant but similar alveolar bone (Figures 7(b), 7(f), 7(c), and 7(g)). This was confirmed by histology with good implant-to-bone contact without any cellular infiltration that would compromise secondary stability (Figure 9(d)). Although we were able to demonstrate no active bone remodeling around the TSAD as evidenced by EPMA and histology, absolute anchorage with TSAD was not achieved. Literature shows evidence of 0–2.7 mm of TSAD displacement with maximum values up to 5.5 mm [31, 32]. Our results showed TSAD displacement of 0.42 mm at 4 weeks and 0.94 mm at 8 weeks. The rate of TSAD displacement was 0.014 mm/day from 0 to 4 weeks and 0.018 mm/day from 4 to 8 weeks. The rate and amount of TSAD displacement was not significant at 4 weeks (*P* ~ 0.057). This failure to achieve absolute anchorage did not prevent using the TSAD as stable anchorage for tooth movement. Statistically significant tooth movement of the right first permanent molar was achieved with 0.62 mm and 1.99 mm of tooth movement at 4 and 8 weeks, respectively, (*P* ~ 0.0003). The rate of tooth movement had an exponential increase from 0.022 mm/day by 4 weeks to 0.048 mm/day by 8 weeks. Both the amount and rate of tooth movement were comparable to published literature [3, 5, 6, 33] Bone remodeling with new bone formation on the tension side of first permanent molar was evident in EPMA analysis with increased strontium deposition (Figure 8(d)) and new bone formation with stretched PDL fibers on histological sections (Figures 9(b) and 9(c)) indicating robust bone remodeling associated with orthodontic tooth movement.

Our study is the first to use TSADs as direct anchorage to facilitate tooth movement overcoming some of the inadequacies associated with previous rodent models of tooth movement. Many of the studies on orthodontic tooth movement in rats used inaccurate, unreliable, and nonphysiologic methods of tooth movement [2–5]. We were able to show that TSADs could be used as a stable anchorage device with significant tooth movement at 4 and 8 weeks, adequate TSAD stability, and maintenance of constant force levels, preventing harmful iatrogenic effects to both the maxillary and mandibular incisors teeth. In previous studies maxillary incisors were used as anchorage to secure the appliance and to prevent appliance loosening during mastication and physiologic eruption [5]; the mandibular incisors were repeatedly ground down resulting in tooth fracture, pain, discomfort, and occasional pulpal exposure of the incisors. Our study allowed for normal physiologic eruption of both the maxillary and mandibular incisors without any iatrogenic trauma; and the orthodontic appliance using TSADs was stable enough to allow normal masticatory process. Most of the previous studies measured tooth movement from 2 to 4 weeks that did not provide clinically significant tooth movement to estimate the stability of the anchorage device [6]. Our study was also able to provide evidence that TSADs could be used as direct anchorage to provide the optimal force levels to allow significant tooth movement of ~2 mm up to 8 weeks. The design of this new model of tooth movement in rats provided evidence that TSADs could be used as effective anchorage devices to provide optimal force levels for clinically and statistically significant tooth movement.

## 5. Conclusions

1. TSADs can be used as a stable anchorage device for OTM in rats.
2. Statistically significant amount of tooth movement was achieved with 0.62 mm at 4 weeks and 1.99 mm at 8 weeks.
3. Success rate of TSADs were 92% at 4 weeks and 80% at 8 weeks.
4. Absolute anchorage was not achieved with secondary TSAD displacement of 0.42 mm at 4 weeks and 0.094 mm at 8 weeks.
